# Polymethylmethacrylat-Zementbeschichtung intramedullärer Implantate: Eine neue Technik für die Revisionschirurgie am Beispiel einer temporären Kniegelenkarthrodese. Videobeitrag

**DOI:** 10.1007/s00132-021-04111-x

**Published:** 2021-05-03

**Authors:** Markus Rupp, Nike Walter, Abdullah Ismat, Volker Alt

**Affiliations:** Klinik und Poliklinik für Unfallchirurgie, Universitätsklinikum Regensburg, Franz-Josef-Strauß-Allee 11, 93053 Regensburg, Deutschland

**Keywords:** Antibiotika, Interne Fixatoren, Polymethylmethacrylat, Reinfektion, *Staphylococcus epidermidis*, Antibiotics, Internal fixators, Polymethyl methacrylate, Reinfection, *Staphylococcus epidermidis*

## Abstract

**Hintergrund:**

Die Beschichtung von intramedullären Stäben mit antibiotikahaltigem PMMA(Polymethylmethacrylat)-Knochenzement sorgt für eine hohe lokale Antibiotikakonzentration und für Stabilität bei noch nicht konsolidierten Frakturen. Allerdings kann sich bei Entfernung des Implantats Knochenzement ablösen und im Markraum von Röhrenknochen verbleiben.

**Fallbeschreibung:**

Eine 56-jährige Patientin litt nach einer periprothetischen Kniegelenkinfektion an einer schmerzhaften Reinfektion mit *Staphylococcus epidermidis*. Es bestand eine Indikation zu einem Ausbau der Prothese.

**Operation:**

Die einliegende Rotations-Scharnier-Prothese wurde nach Weichteildebridement und Synovektomie entfernt. Für eine temporäre Arthrodese wurden als intramedulläre Implantate Humerusnägel verwendet, die mit antibiotikahaltigem PMMA-Zement beschichtet waren. Um ein Ablösen des Knochenzements bei der Implantatentfernung und einen Verbleib von Zementresten im Knochen zu verhindern, wurden die Humerusnägel mit Cerclagedraht armiert. Das so beschichtete Implantat wurde dann „pressfit“ in den Markraum implantiert.

**Video online:**

Die Online-Version dieses Beitrags (10.1007/s00132-021-04111-x) enthält ein Video zur PMMA-Zementbeschichtung bei intramedullären Implantaten.

## Abstract (en)

**Background:**

The coating of intramedullary rods with polymethyl methacrylate (PMMA) bone cement containing antibiotics ensures a high concentration of antibiotics locally and stability in fractures that are not yet consolidated. However, bone cement can detach when the implant is removed and remain in the medullary cavity of the long bones.

**Case report:**

After a periprosthetic knee infection, a 56-year-old patient suffered from a painful reinfection of *Staphylococcus epidermidis*. This was an indication for removal of the prosthesis.

**Operation:**

After soft-tissue debridement and synovectomy, rotating hinge prosthesis that had been inserted was removed. Humeral nails coated with PMMA cement containing antibiotics were used as temporary intramedullary implants. To prevent the bone cement from detaching when the implant was removed and cement residue from remaining in the bone, the humeral nails were reinforced with cerclage wire. The coated implant was then press fit into the medullary cavity.

## Hintergrund

Seit der Einführung von antibiotikahaltigem PMMA-Knochenzement zur Verringerung der Rate an periprothetischen Infektionen in der Endoprothetik durch Buchholz und Engelbrecht Ende der 1960er-Jahre hat sich der Einsatz von PMMA-Zement als lokaler Antibiotikaträger zur Infektprophylaxe und Infekttherapie in Orthopädie und Unfallchirurgie etabliert [1–3]. Neben den im Handel erhältlichen Gentamicin-haltigen PMMA-Ketten, wurde die lokale Applikation von PMMA zur Ummantelung von Osteosynthesematerialien beschrieben [4, 5]. Die Ummantelung von intramedullären Stäben mit antibiotikahaltigem PMMA-Knochenzement bietet verschiedene Vorteile. Neben hohen lokalen Antibiotikakonzentrationen können diese bei noch nicht konsolidierten Frakturen Stabilität gewährleisten und hierdurch eine frakturassoziierte Infektion nach chirurgischem Debridement zur Ausheilung bringen. Die erzielte Stabilität kann zudem eine frühzeitige Belastung der Extremität ermöglichen. Indes bieten PMMA-Zement-beschichtete Implantate eine Alternative zur sonst, in vielen Fällen notwendigen, externen Stabilisierung. Nach periprothetischen Infektionen kann die Zementbeschichtung von intramedullären Stäben zur Herstellung von temporären Kniearthrodesen dienen [6]. Unabhängig von der Indikation hat der Gebrauch von PMMA-beschichteten Stäben oder Marknägeln verschiedene Limitationen, die sich von Schwierigkeiten der Herstellung bis hin zu Problemen bei der Entfernung der Implantate im Rahmen von Folgeeingriffen erstrecken. Bei Letzteren stellt das Ablösen des PMMA-Knochenzementes vom Implantat und somit das Verbleiben des Zementes im Markraum langer Röhrenknochen ein Problem dar. Gerade die Entfernung von tief im Markraum befindlichen Zementresten kann operativ aufwendig und zeitraubend sein. Ein Verbleib von biofilmhaltigen infizierten Zementresten kann indes als Nidus für eine Reinfektion angesehen werden. Die hier vorgestellte Technik der Bewehrung von PMMA-Knochenzement im Rahmen der Ummantelung von Marknägeln oder Stäben kann Problemen bei der Entfernung von PMMA-Zement-beschichteten Stäben vorbeugen.

## Fallbeschreibung

**Figure 1:**
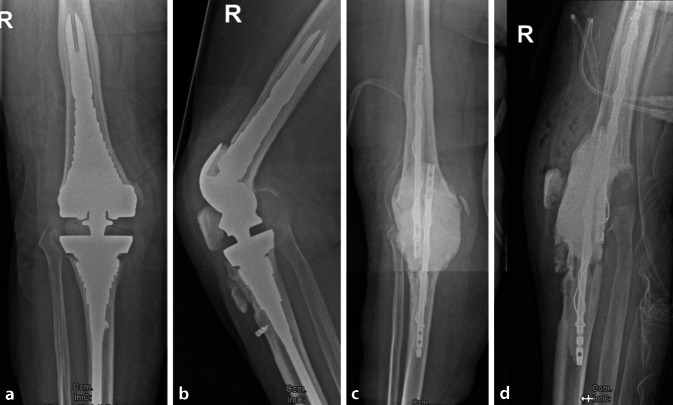

In dem im Video dargestellten Fall handelt sich es um eine 56-jährige Patientin, die an einer Reinfektion nach periprothetischer Kniegelenkinfektion leidet. Die Patientin erhielt im Rahmen eines zweizeitigen Wechsels in einem auswärtigen Krankenhaus 2011 eine Reimplantation einer Rotationsscharnierprothese (S-ROM®, Depuy-Synthes, Warsaw, IN, USA) am rechten Kniegelenk (Abb. 1a, b). Die Patientin berichtete bei Vorstellung in unserer Klinik von rezidivierenden, bereits monatelangen Schmerzen im rechten Kniegelenk. Nach Patellasehnenruptur und Refixation im Jahre 2018 besteht ein deutliches Beugedefizit (Extension/Flexion 0‑0-80°). Eine Rötung bestand seit einer Woche vor dem durchgeführten Revisionseingriff. Durch Punktion des Kniegelenkes konnte *Staphylococcus epidermidis* nachgewiesen werden. Bei einer Gesamtzellzahl von 15680 Zellen/µl und einem Granulozytenanteil von 92,9 % wurde von einem Rezidiv einer periprothetischen Infektion ausgegangen und die Indikation zum Ausbau der Knieprothese im Rahmen eines zweizeitigen Vorgehens gestellt. Im Videobeitrag wird die Technik der temporären Arthrodese vorgestellt, wobei der Fokus hierbei auf der Beschichtungstechnik der verwendeten Marknägel durch Bewehrung des PMMA-Knochenzementes mit Cerclagedraht liegt.

## Operationsablauf

### Prothesenausbau und Debridement

Das Kniegelenk wird über einen präpatellaren Zugang eröffnet. Nach Eröffnen des Gelenkes erfolgt das Weichteildebridement mit vollständiger Synovektomie sowie das Entfernen der einliegenden Rotations-Scharnier-Prothese. Die Femurkondylen und das Tibiaplateau werden von Zementresten befreit und debridiert. Anschließend werden die Markräume aufgebohrt, femoral bis zu einer Größe von 14 mm, tibial kann der Markraum bis zu einem Durchmesser von 12,5 mm aufgebohrt werden. Der Situs wird mit insgesamt 9 l Kochsalzlösung durch Jet-Lavage ausgespült. Anschließend wird Granudacyn-Wundspüllösung (Mölnlycke Health Care GmbH, Düsseldorf, Deutschland) in den Situs appliziert.

### Implantate zur Beschichtung mit antibiotikahaltigem PMMA-Knochenzement

Als intramedulläre Implantate werden im vorgestellten Fall Humerusnägel verwendet (T2, Stryker, Duisburg, Deutschland). Grundsätzlich lassen sich hierfür auch andere Osteosynthesematerialien verwenden, wie dies bereits in der Literatur beschrieben wurde (Kirschner-Drähte, Längsverbinder, Gewindestäbe, Karbonstäbe) [5, 7]. Die vorgestellte Technik der Verwendung von Verriegelungsmarknägel bietet den Vorteil, dass das eingebrachte Implantat Bohrlöcher und zusätzlich ein Gewinde am proximalen Ende zur Montage eines Extraktionsinstrumentariums besitzt. Dies kann bei erschwerter operativer Entfernung von entscheidendem Vorteil gegenüber beispielsweise glatten Längsverbindern sein. Die Verwendung von Humerusnägeln an der unteren Extremität soll indes nicht weiter irritieren. Auf der einen Seite muss der Durchmesser des metallischen Implantates bei Markraumdurchmessern von, wie im beschriebenen Fall, bis zu 14 mm gerade noch schmal genug sein, um eine vollständige Ummantelung zu gewährleisten. Auf der anderen Seite birgt ein zu dünn gewähltes metallisches Implantat die Gefahr eines Materialbruches mit einer notwendigen vorzeitigen, nicht geplanten operativen Revision [8]. Die Erfahrung der Autoren zeigt, dass Marknägel mit einem Durchmesser von 7 mm ausreichend Stabilität bieten. Ein Implantatbruch konnte bisher in der eigenen Patientenpopulation nicht beobachtet werden.

Die Materialkosten belaufen sich pro Humerusnagel auf 443 €. Ein zu Humerusnägeln alternativer Längsverbinder aus dem Wirbelsäuleninstrumentarium, welcher jedoch kein Gewinde für ein Extraktionsinstrumentarium oder Verriegelungslöcher besitzt, würde ca. 200 € kosten. Erwägt man die Implantation mobiler Spacer, in Fällen bei denen die knöcherne Defektsituation dies erlaubt, betragen die Materialkosten etwa 440 € für die zu verwendeten Formschalen. Beim Vergleich der Materialkosten ist zu beachten, dass diese, abhängig von hausinternen Konditionen bei verschiedenen Herstellern, deutlich variieren können. Die verwendete Zementmenge ist für alle Behandlungsalternativen vergleichbar, sodass hier kein nennenswerter, preislicher Unterschied resultiert. Bei einem DRG-Erlös von 14.083,57 € (DRG I04Z), der beim Einsatz mobiler Spacer oder einer temporärer Arthrodese identisch ist, ist die Verwendung von Humerusnägeln für eine temporäre Arthrodese aus Sicht der Autoren aufgrund der leichteren Entfernung der Nägel im Rahmen der Folgeoperation zu vertreten.

### Optimierung der PMMA-Zementbeschichtung durch Cerclagedraht

**Figure 2:**
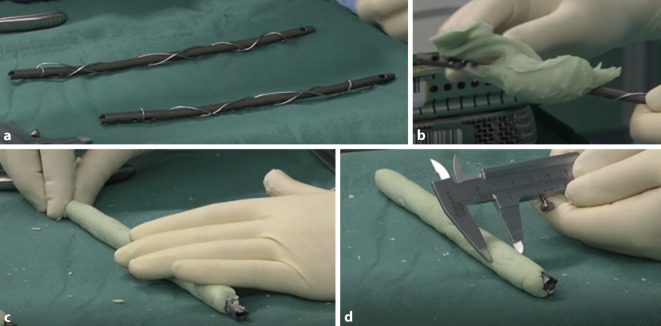

Dem Problem des Ablösens des Zementes vom metallischen Implantat im Rahmen der Implantatentfernung wird durch die Verwendung eines zusätzlichen Cerclagedrahtes bei der Beschichtung von Marknägeln vorgebeugt (Abb. 2a). Diese Technik ähnelt der im Bauwesen als Bewehrung bekannte Verstärkung von Beton. Durch Einbringen von Stahl in Beton werden mechanischen Eigenschaften verbessert, was bereits seit langem eine übliche Technik ist [9]. Der Cerclagedraht wird, beginnend an einem Verriegelungsloch, von proximal nach distal und wieder nach proximal verspannt (Video ab 00:28 min). Als PMMA-Knochenzement wird Copal® G + C-Zement (Heraeus) verwendet. Dieser beinhaltet, wie die Buchstaben G + C implizieren Gentamicin und Clindamycin, jeweils 1 g auf 40 g PMMA-Knochenzement. Bei im vorgestellten Fall benutzten T2-Humerusnägeln der Länge 240 mm und 220 mm müssen 80 g Copal®-Zement angemischt werden, um eine vollständige Beschichtung gewährleisten zu können. Nach 3 min des Aushärtens erreicht der PMMA-Knochenzement eine Konsistenz, die es zulässt, den mit Cerclagedraht umwickelten Marknagel zu umhüllen (Abb. 2b). Damit eine gleichmäßige Beschichtung des Marknagels erzielt werden kann, wird der Marknagel samt Zement auf dem Operationstisch gerollt bis ein gleichmäßiger und glatter Zementmantel geschaffen ist (Abb. 2c, Video ab 02:17 min). Mit der Schieblehre wird bei noch verformbarem PMMA-Knochenzement der Durchmesser an verschiedenen Stellen des nun beschichteten Marknagels gemessen (Abb. 2d, Video ab 04:28 min). Hier sollte unbedingt darauf geachtet werden, dass der vorher erzielte Markraumdurchmesser nicht überschritten wird. Aus eigener Erfahrung lässt sich ein beschichtetes Implantat mühelos „pressfit“ in den Markraum implantieren, wenn die gemessenen Durchmesser dem aufgebohrten entsprechen oder minimal kleiner sind. Bei der Implantation des PMMA-Knochenzement-beschichteten Stabes ist darauf zu achten, dass dieser im vollständig ausgehärteten Zustand implantiert wird. In der Regel sind 12 min Aushärtezeit nötig. Ein noch weicher Zement sollte nicht implantiert werden, da ein Hineinpressen des PMMA-Zementes in die Spongiosa resultieren kann, was eine Entfernung eines PMMA-beschichteten Implantates deutlich erschwert. Zudem sollte beim Einschlagen des beschichteten Marknagels darauf geachtet werden, dass es nicht zu einer Fraktur des betreffenden Knochens kommt, was – ähnlich wie beim Einbringen zementfreier Prothesenschäfte in der Endoprothetik – durchaus möglich ist.

### Temporäre Kniegelenkarthrodese – Pseudofusion

Die im Markraum platzierten PMMA-Knochenzement-beschichteten Stäbe werden in der gelenkseitigen knöchernen Defektzone überlappend platziert (Video ab 06:34 min). Das Kniegelenk wird in etwa 10–15° Beugestellung und in physiologischer normvalgischer Beinachse gehalten resp. gelagert. Die Defektzone wird im vorliegenden Fall mit 120 g Copal®-Zement, welchem 6 g Vancomycin-Pulver beigemischt wurde, aufgefüllt (Video ab 06:45 min). Hierbei muss auf das möglichst dorsale Umfassen der in der Defektzone einliegenden proximalen, sich überlappenden Stabenden geachtet werden. Das Umschließen des später ausgehärteten Zementes ist für eine belastbare temporäre Arthrodese essenziell. Eine weitere mechanische Verbindung zwischen beiden ummantelten Marknägeln ist bei ausreichender Überlappung und Umfassen der Marknägel durch die Zementplombe nicht weiter nötig (Abb. 1c, d). Die temporäre Kniegelenkarthrodese, die im vorliegenden Fall aufgrund der Defektgröße gegenüber einem mobilen Spacer bevorzugt wird, ermöglicht es den Patienten das Leben mit Kniegelenkarthrodese zu erfahren und somit später die mögliche Therapieentscheidung pro Kniegelenkarthrodese durch Kenntnis der Lebensumstände besser mitzutragen. Eine Aussage über eine Limitierung der Technik hinsichtlich der zu überbrückenden Defektgröße ist bisher noch nicht möglich und Gegenstand zukünftiger Untersuchungen.

## Fazit für die Praxis

- Die Bewehrung von PMMA(Polymethylmethacrylat)-Knochenzement-beschichteten Implantaten ist eine einfache und kostengünstige Methode, um ein Ablösen des Knochenzementes beim Entfernen des Implantates im Rahmen einer Revisionsoperation zu verhindern.
- Bewehrte PMMA-Knochenzement-beschichtete Implantate lassen sich sowohl für temporäre Arthrodesen, artikulierende intramedullär verankerte Spacer als auch für eine intramedulläre Stabilisierungen langer Röhrenknochen verwenden.
- Mangels randomisierter Vergleichsstudien kann eine Überlegenheit von bewehrten gegenüber unbewehrten Implantaten hinsichtlich postoperativer Komplikationen noch nicht nachgewiesen werden.

## Supplementary Information





## Abkürzungen

DRG: „Diagnosis Related Groups“
PMMA: Polymethylmethacrylat
